# Studies evaluating of health interventions at schools: an integrative literature review

**DOI:** 10.1590/1518-8345.2463.3008

**Published:** 2018-07-16

**Authors:** Eliabe Rodrigues de Medeiros, Danielle Gonçalves da Cruz Rebouças, Alany Carla de Sousa Paiva, Camila Priscila Abdias do Nascimento, Sandy Yasmine Bezerra e Silva, Erika Simone Galvão Pinto

**Affiliations:** 1 Doctoral student, Departamento de Enfermagem, Universidade Federal do Rio Grande do Norte, Natal, RN, Brazil. Bolsista do Conselho Nacional de Desenvolvimento Científico e Tecnológico (CNPq), Brazil.; 2 Cardiology and Hemodynamics Specialist, Specialization student in Public Health: Oncology Nursing, Escola da Assembleia Legislativa do Rio Grande do Norte, Natal, RN, Brazil.; 3 Occupational Health Nursing Specialist, Specialization student in Higher Education Teaching, Escola de Saúde, Universidade Potiguar, Natal, RN, Brazil.; 4 Emergency and Trauma Nursing Specialist, Specialization student in Public Health, Faculdade de Saúde Pública, Universidade de São Paulo, São Paulo, SP, Brazil.; 5 Master’s student, Departamento de Enfermagem, Universidade Federal do Rio Grande do Norte, Natal, RN, Brazil.; 6 PhD, Professor Adjunto, Departamento de Enfermagem, Universidade Federal do Rio Grande do Norte, Natal, RN, Brazil.

**Keywords:** Health, Education, Health Services Research, School Health Services, Program Evaluation, Evaluation Studies

## Abstract

**Objective:**

to identify and analyze the available evidence on the strategies used in the studies evaluating health interventions at school.

**Method:**

this is an integrative review searching in LILACS, CINAHL, CUIDEN, ScienceDirect, and PubMed. From the pre-defined inclusion and exclusion criteria, there were 121 articles chosen to compose the sample.

**Results:**

english studies (97.5%), with a quantitative approach (80.2%), related to the interventions carried out in the Region of the Americas (54.6%) and the European Region (23.1%) predominated. For the most part, they are interventions as programs (70.2%), interested in evaluating results (73.5%) from the value judgment (83.4%). Prevalence of interventions focused on efficacy, effects or impact, and activities carried out on interventions were focused on physical activity, healthy eating, sexual and reproductive health, mental health, and use of tobacco, alcohol, and other drugs. They are worked through activities of clinical monitoring, health promotion and disease prevention.

**Conclusion:**

the evidence indicates that the evaluations of health interventions in the school focus the results produced in programs through the judgment of value. The topics most addressed were healthy eating, physical activity, prevention of alcohol and other drugs, among others.

## Introduction

The offering of equal and equitable education across the globe has been one of the flags raised by international organizations in encouraging children and adolescents to be enrolled in schools. Also, it is also necessary to develop health interventions so these individuals do not have the educational process interrupted under the influence of diseases and other health problems^1^.

In these spaces, it is possible to contribute to the development of learners through interventions that subsidize educational success through the provision of care that may not be experienced at school. The lack of school health interventions together with the compromising situations (diseases and/or aggravations) prevalent in schools make the learning process difficult, where it is necessary to provide continuous care to the health of the children and adolescents in the school environment^2^.

In 1995, the World Health Organization (WHO) encouraged the development of the global strategy of Health Promoting Schools, characterized by the constant search to strengthen the capacity to promote a healthy life, with an incentive to learn and working conditions, to respond health needs of the school community^3^.

Under this influence and based on the Ottawa Charter and as a result of the First International Conference on Health Promotion held in 1986, discussion groups were set up to encourage the development of health promotion strategies in school settings, such as the European Network of Health Promoting Schools and the Latin American Network of Health Promoting Schools^4^.

This fact contributed to creating several experiences all over the planet. However, because they are countries with diverse economic, political, cultural, and other contextual characteristics, it is necessary to consider that these interventions can be executed based on different objectives. Also, difficulties and challenges can be found in their implementations, requiring the need to develop evaluative processes that seek to improve them.

This is possible since the evaluation has been considered as an important tool to aid the management of health interventions in the search for better answers to services resulting from improvement, resolution and better quality^5^.

The evaluations of the health interventions at school have been carried out and involve specific topics such as prevention of depression and anxiety^6^, the offering of physical activities to students^7^ and drug prevention among schoolchildren^8^. No studies have been found that synthesize comprehensively the evaluation of health interventions in school in the world and its different themes.

The purpose of this study is to synthesize the studies that carried out evaluations of health interventions at school, so their strategies and the diversity of interventions directed to this space can be identified. This will contribute to other research being carried out from the findings presented here.

The study advances knowledge as it presents the evidence on strategies used in evaluations of health interventions at school, enabling other health interventions at school to be evaluated as well.

Evaluations should be carried out by management and by health and education professionals to contribute to the promotion of health at school. It is observed by the activities developed in the daily life that the nurse performs interventions in the school environment in a continuous way.

The actions or health activities carried out in the school, such as policies, programs, projects, services, and systems, were considered an intervention. Evaluations are understood as the decision-making processes that aid in the improvement of the health interventions in the school. Therefore, there is an intimate relationship between the two, since it is not possible to evaluate interventions without knowing their organization.

In view of this information, the objective is to identify and analyze the available evidence on the strategies used in the evaluation studies of health interventions in the school.

## Method

The integrative review of the literature was the methodological strategy chosen to respond to the proposed objective. It consists of a broad methodology of research analysis to synthesize knowledge about a given topic. The elaboration of a study of this nature runs through five stages: identification of the problem and elaboration of the guiding question; search for studies in the literature; evaluation of data found in the studies; data analysis with synthesis and their conclusions and the presentation of the integrative review^9^.

A protocol was built by the researchers to guide the construction of the study. The guiding question was elaborated with the help of the PICO strategy^10^ (P: health interventions at school, I: evaluation studies, C: not applicable, O strategies used). What evidence is available about the strategies used in the evaluation studies of health interventions at school?

The search for the studies was carried out in May 2017 through the Portal of Journals of Capes with access through the Federated Academic Community (CAFe) in which the researchers are linked. The studies were selected in the electronic databases of *Literatura Latino-Americana e do Caribe em Ciências da Saúde* (LILACS), Cumulative Index to Nursing and Allied Health Literature (CINAHL), *Base de Datos Bibliográfica de la Fundación Index* (CUIDEN), ScienceDirect and US National Library of Medicine (PubMed).

To proceed with the search, descriptors in Health Sciences (DeCS) were used for the databases in Portuguese and Spanish and the corresponding Medical Subject Headings (MeSH), for the search in the electronic bases in English. The Boolean operators AND and OR were used to cross the descriptors as follows: “Serviços de Saúde Escolar *AND* Avaliação de Programas e Projetos de Saúde *OR* Avaliação de Serviços de Saúde”, “*Servicios de Salud Escolar AND Evaluación de Programas y Proyectos de Salud OR Evaluación de Servicios de Salud*” e “*School Health Services AND Program Evaluation OR Health Services Research*”.

The inclusion criteria consisted of articles published in the last five years, available for free access in full in English, Spanish and Portuguese, which addressed the proposed theme. Editorials, letters to the editor, review studies, theses, dissertations, articles and studies that did not correspond to the relevant theme within the scope of the review were excluded.

**Figure 1:**
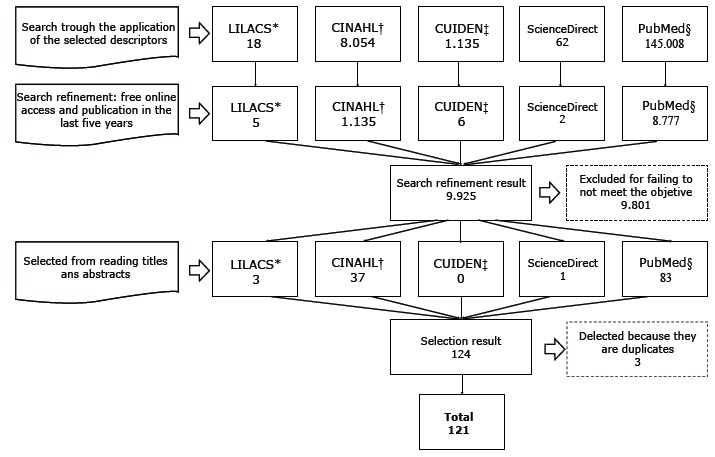
Flowchart of identification of the selection process of the selected studies to compose the integrative review. Natal, RN, Brazil, 2017

The research and selection of the studies were carried out by two researchers, simultaneously. When a situation of divergence happened, a consensus was sought with the participation of an auxiliary researcher. The process of searching and selecting the studies followed the PRISMA recommendations^11^ and is represented in Figure 1.

* LILACS: Literatura Latino-Americana e do Caribe em Ciências da Saúde † CINAHL: *Cumulative Index to Nursing and Allied Health Literature* ‡ CUIDEN: *Base de Datos Bibliográfica de la Fundación Index* § PubMed: *US National Library of Medicine*

The initial search in each database using the search term crossings was preceded by its refinement (free online access and publication in the last five years) in each of the electronic bases from the use of the available tools in the electronic portals. In the sequence, the titles and abstracts were read, making a quantitative of 124 studies in all the electronic bases. Also, repeated studies were excluded, where the final sample resulted in 121 articles.

The analysis of the selected studies was performed based on the pre-selected variables in the protocol construction. To organize them, a spreadsheet created in Microsoft Excel Software was used, containing the following items: database, journal, language, year of publication, methodological approach used, geographical location where the intervention was performed according to WHO classification, type (structure, process and outcome) according to Avedis Donabedian’s theoretical reference^12^, level of evaluation (description, measurement, judgment, negotiation) according to the classification identified in Furtado’s study^13^, type of evaluation, besides description of the themes and characteristics of the intervention.

The analysis and discussion of the results were based on the national and international literature on school health and health evaluation.

## Results

The characterization of the selected studies^14^ ^-^ ^134^ included in the integrative review is presented below. In Table 1, information regarding the year and language of publication, the approach used in these surveys and the place of execution of the interventions are observed.

**Table 1:** Characterization of the studies regarding the year, language, approach and place of intervention. Natal, RN, Brazil, 2017

Variable	N*	%^†^
Year of publication		
2013	32	26.5
2014	32	26.5
2015	34	28.0
2016	19	15.7
2017	4	3.3
Language		
English	118	97.5
Spanish	3	2.5
Type of research approach		
Quantitative	97	80.2
Qualitative	10	8.2
Quantitative and Qualitative	14	11.6
Regions of interventions		
African Region	5	4.1
Region of the Americas	66	54.6
Southeast Asia Region	7	5.8
European Region	28	23.1
Eastern Mediterranean Region	4	3.3
Western Pacific Region	11	9.1
Total	121	100.0

*N: number; †%: percentage

It should be noted that the lower percentage of studies published in 2017 is explained by the fact that the data collection was carried out with the year still in progress.

Information regarding the classification of the type of intervention, proposed evaluative dimension and level of evaluation performed are shown in Table 2.

**Table 2:** Characterization of the studies regarding the type of intervention, dimension evaluated and level of evaluation performed. Natal, RN, Brazil, 2017

Variable	N*	%^†^
Type of Intervention		
Policy	6	5.0
Program	85	70.2
Project	19	15.7
Service	9	7.4
System	2	1.7
Dimension evaluated		
Structure	2	1.7
Process	24	19.8
Result	89	73.5
Structure and Result	2	1.7
Process and Result	3	2.5
Structure, Process, and Result	1	0.8
Level of Evaluation		
Description	11	9.1
Measure	3	2.5
Judgment	101	83.4
Negotiation	6	5.0
Total	121	100.0

*N: number; †%: percentage

**Figure 2:**
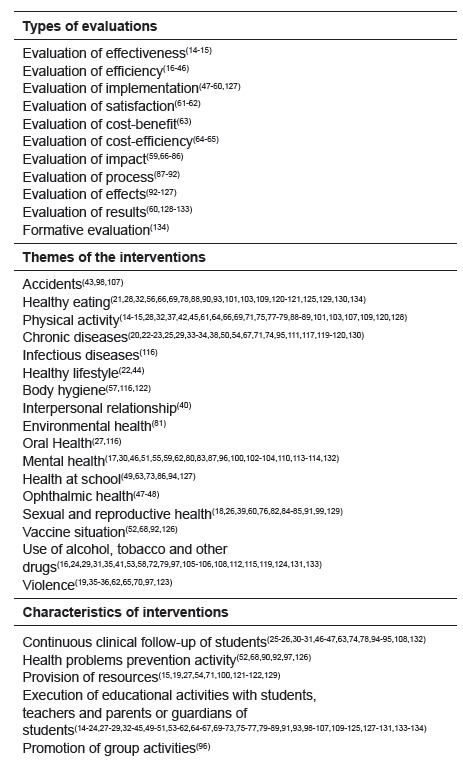
Characterization of studies evaluating health interventions in school. Natal, RN, Brazil, 2017

It was also possible to describe the interventions regarding the themes, characteristics, and typologies of evaluations proposed in their methodologies, as presented in Figure 2.

Thus, the data show that the evaluation of effects, efficacy, and impact was the most prevalent among the types of evaluation found.

It is observed that interventions of various themes have been carried out in the school environment, whether they are in a broader theme, such as activities that aim to encourage healthier lifestyles or more specific themes such as those that prevent accidents from occurring among learners.

These themes are worked with the school community through strategies that address the continuous clinical follow-up of learners^25^ ^-^ ^26^ ^,^ ^30^ ^-^ ^31^ ^,^ ^46^ ^-^ ^47^ ^,^ ^63^ ^,^ ^74^ ^,^ ^98^ ^-95,^ ^108^ ^,^ ^132^, through activities to prevent health problems^52^ ^,^ ^68^ ^,^ ^90^ ^,^ ^92^ ^,^ ^97^ ^,^ ^126^, availability of resources in school spaces as a way of encouraging changes in habits^15^ ^,^ ^19^ ^,^ ^27^ ^,^ ^54^ ^,^ ^71^ ^,^ ^100^ ^,^ ^121^ ^-^ ^122^ ^,^ ^129^, educational activities with students and other members of the school community^(14-24,27-29,32-45,49-51,53-62,64-67,69-73,75-77, 79-89,91,93,98-107,109- 125,127-131,133-134)^ and group activities^96^.

## Discussion

The high number of studies that integrated the integrative review shows the possible concern of the researchers in offering quality interventions in the school environment, which can be done when submitting them to the evaluation processes. However, a smaller number of publications were observed in the last two years of the analysis, which can be explained by the inclusion of a year still in progress during the review period.

The predominance of studies published in the English language reflects the fact that this is a widely disseminated language in the world. Therefore, it was considered by the scientific community as a universal language to contribute to the dissemination of publications in the world and to promote better access^135^.

When analyzing the types of approaches used in evaluative surveys of health interventions at school, it was identified that most of them used exclusively the quantitative approach. In contrast, there were studies that proposed qualitative analysis. However, this type of research requires the appropriation of several means to understand the problems, which is possible with the integration of these two types of approaches^136^.

The observation that there was a greater predominance of studies published in the Region of the Americas, mainly in the United States of America, and in the European Region, shows the understanding that the largest world economic powers are located there, and can consequently have greater investments in the execution of searches. In contrast, the regions in which the nations with the lowest human development indicators are found are also those with a reduced number of health interventions in school.

According to the classification of the interventions proposed for analysis, it was identified that most of them refer to programs to improve students´ health. On the other hand, there is a discrete presence of interventions classified as systems that offer the search for health care to learners.

Prevalently, it was identified that most evaluative research sought to carry out evaluations focusing on the results of the interventions. It is important to emphasize the need to consider the development of these evaluations so they do not only focus on the presence or absence of the results brought about by the intervention, but also try to understand the factors that influenced the process^137^. It is also considered that, although studies are usually found that deal with outcome evaluations, the structure and process can influence the presentation of what is expected of these interventions^12^. Therefore, it is important to emphasize the equal importance of these dimensions in proposals for the execution of activities directed at school spaces.

Considering the level of evaluation of the research carried out, a large number of studies with an evaluation at the judgment level were found. Thus, evaluations of health interventions at school exceeded descriptive and measurement evaluations when judging value. However, there is still a small number of evaluations that propose a process of negotiation between those involved in the school environment^138^.

Thus, although the nomenclatures attributed to the evaluations are diversified, they can be performed identically. Nevertheless, it is noted that these types of proposed evaluations aim for interventions to perform the search for better results^139^.

The evaluations found may also influence the evaluation levels identified from the historical generations. Thus, in its first generation, the evaluations were descriptive. In the second generation of evaluations, there was research that sought to measure performance. The third generation has evaluations that try to judge the merits of the interventions. In the fourth generation, there is an evaluation that advocates the participatory negotiation process among the different participants in the intervention^13^.

The fourth-generation evaluation can be carried out in school health interventions, since they have activities that allow the participation not only of the students but also of teachers, other school workers, health professionals, parents or responsible for the students in their proposals, as well as representatives of the community in which the schools are inserted^140^.

Regarding the themes in which these interventions were proposed, similar results were observed in a study carried out in the United Kingdom on the structures of the World Health Promoting Schools^141^. The following themes of interventions prevailed: physical activities, healthy eating, sexual and reproductive health, mental health and tobacco use, alcohol and other drugs.

As for the characteristics of these interventions, it was found that besides to continuous follow-up of students, preventive activities, health promotion with the school community and use of talk circles, there is the availability of resources to encourage a change of habits. This last characteristic corroborates with a research carried out in Canada, which refers to the presence of objects and food to encouraging the promotion of healthy eating and physical activities^142^.

The diversity of the themes and the characteristics related to health interventions at the school evaluated is essential in changing habits for the life of the students and the community where they live. This is identified in a study that shows that, these activities are of great importance to the health of the population although they seem small actions in face of the diversity of needs found^141^.

It should be emphasized that the limitations of this research were related to the high number of studies eligible for an integrative review, although the criteria chosen were delimited. Also, it is observed that the use of more than one researcher for data analysis may influence the presented results, although they were able to perform this activity.

## Conclusions

The evidence found in the studies show that school health programs are the most commonly evaluated interventions, especially at the value judgment level. These studies are mainly focused on evaluating the results produced by the interventions, corroborated in the typologies of impact, effects, efficacy, and results.

The themes of the studies are related to healthy eating, physical activity, mental, sexual and reproductive health, as well as prevention of the consumption of alcohol, crack and other drugs. They are implemented through strategies of continuous clinical monitoring, health promotion, disease prevention and health problems, and group activities involving students and other members of the school community.

## References

[1] United Nations Educational, Scientific and Cultural
Organization (2017). Reducing global poverty through universal primary and secondary
education.

[2] Chidiebere ODI, Thomas UO, Joy E, Stanley OK, Ikenna NK, Uchenna E (2016). The Status of School Health Services A Comparative Study of
Primary Schools in a Developing Country. Am J Public Health Res.

[3] World Health Organization Health Promoting School: an effective approach for early action on NCD
risk factors.

[4] Silva CS, Bodstein RCA (2016). A theoretical framework on intersectoral practice in School
Health Promotion. Cienc Saúde Coletiva.

[5] Tanaka OY, Drumond M, Cristo EB, Spedo SM, Pinto NRS (2015). Cluster analysis as a tool for management improvement in the
SUS. Saúde Soc.

[6] Werner-Seidler A, Perry Y, Calear AL, Newby JM, Christensen H (2017). School-based depression and anxiety prevention programs for young
people A systematic review and meta-analysis. Clin Psychol Rev.

[7] Mei H, Xiong Y, Xie S, Guo S, Li Y, Guo B (2016). The impact of long-term school-based physical activity
interventions on body mass index of primary school children - a
meta-analysis of randomized controlled trials. BMC Public Health.

[8] Flynn AB, Falco M, Hocini S (2015). Independent Evaluation of Middle School-Based Drug Prevention
Curricula A Systematic Review. JAMA Pediatr.

[9] Hopia H, Latvala E, Liimatainen L (2016). Reviewing the methodology of an integrative
review. Scand J Caring Sci.

[10] Santos CMC, Pimenta CAM, Nobre MRC (2007). The PICO strategy for the research question construction and
evidence search Rev. Latino-Am. Enfermagem.

[11] Moher D, Liberati A, Tetzlaff J, Altman DG, The Group PRISMA (2009). Preferred Reporting Items for Systematic Reviews and
Meta-Analyses The PRISMA Statement. PLoS Med.

[12] Donabedian A (2005). Evaluating the Quality of Medical Care. Milbank Q.

[13] Furtado JP (2001). A Constructivist method for health evaluation. Cienc Saúde Coletiva.

[14] Seo DC, King MH, Kin N, Sovinski D, Meade R, Lederer AM (2013). Predictors for moderate- and vigorous-intensity physical activity
during an 18-month coordinated school health intervention. Prev Med.

[15] Cradock AL, Barrett JL, Carter J, McHugh A, Sproul J, Russo ET (2014). Impact of the Boston Active School Day policy to promote physical
activity among children. Am J Health Promot.

[16] Turhan A, Onrust SA, Klooster PM, Pieterse ME (2017). A school-based programme for tabacco and alcohol prevention in
special education effectiveness of the modified 'healthy school and drugs'
intervention and moderation by school subtype. Addiction.

[17] Onge JRS, Stephenson R, Kumar BS (2016). Validation of the FRIENDS Anxiety Prevention Program for Children
in Canada. Can J Commun Ment Health.

[18] Loosier PS, Doll S, Lepar D, Ward K, Gamble G, Dittus PJ (2016). Effectiveness of an Adaptation of the Project Connect Health
Systems Intervention Youth and Clinic-Level Findings. J Sch Health.

[19] Avci D, Kelleci M (2016). Effects of the Anger Coping Programme based on cognitive
behavioural techniques on adolescents' anger, aggression and psychological
symptoms. Int J Nurs Pract.

[20] Lwin MO, Malik S, Chua TSJ, Chee TS, Tan YS (2016). Intergenerational transfer of blood pressure knowledge and
screening a school-based hypertension awareness program in
Singapore. Glob Health Promot.

[21] Zhou WJ, Xu XL, Li G, Sharma M, Qie YL, Zhao Y (2016). Effectiveness of a school-based nutrition and food safety
education program among primary and junior high school students in
Chongqing, China. Glob Health Promot.

[22] Bhave S, Pandit A, Yeravdekar R, Madkaikar V, Chinchwade T, Shaikh N (2016). Effectiveness of a 5-year school-based intervention programme to
reduce adiposity and improve fitness and lifestyle in Indian children; the
SYM-KEM study. Arch Dis Child.

[23] Tatsuo A, Chiaki Y, Yuki S, Yuya S, Yasuteru I, Akiko I (2014). Stroke Education Program of Act FAST for Junior High School
Students and Their Parents. J Stroke Cerebrovasc Dis.

[24] Giuseppe G, Giulia C, Sandra B, Marco T, Claudia M, Simone S (2014). Effectiveness of a school-based multi-component smoking
prevention intervention The LdP cluster randomized controlled
trial. Prev Med.

[25] Rito AI, Carvalho MA, Ramos C, Breda J (2013). Program Obesity Zero (POZ) - a community-based intervention to
address overweight primary-school children from five Portuguese
municipalities. Public Health Nutr.

[26] Griswold CH, Nasso JT, Swider S, Ellison BR, Griswold DL, Brooks M (2013). The Prenatal Care at School Program. J Sch Health.

[27] Matsuyama Y, Aida J, Taura K, Kimoto K, Ando Y, Aoyama H (2016). School-Based Fluoride Mouth-Rinse Program Dissemination
Associated With Decreasing Dental Caries Inequalities Between Japanese
Prefectures An Ecological Study. J Epidemiol.

[28] Maatoug J, Msakni Z, Zammit N, Bhiri S, Harrabi I, Boughammoura L (2015). School-Based Intervention as a Component of a Comprehensive
Community Program for Overweight and Obesity Prevention, Sousse, Tunisia,
2009-2014. Prev Chronic Dis.

[29] Brinker TJ, Stamm-Balderjahn S, Seeger W, Klingelhöfer D, Groneberg DA (2015). Education Against Tobacco (EAT) a quasi-experimental prospective
evaluation of a multinational medical-student-delivered smoking prevention
programme for secondary schools in Germany. BMJ Open.

[30] Langley AK, Gonzalez A, Sugar CA, Solis D, Jaycox L (2015). Bounce back Effectiveness of an elementary school-based
intervention for multicultural children exposed to traumatic
events. J Consult Clin Psychol.

[31] Strøm HK, Adolfsen F, Handegård BH, Natvig H, Eisemann M, Martinussen M (2015). Preventing alcohol use with a universal school-based
intervention: results from an effectiveness study. BMC Public Health.

[32] Kilanowski JF, Gordon NH (2015). Making a Difference in Migrant Summer School Testing a Healthy
Weight Intervention. Public Health Nurs.

[33] Kintner EK, Cook G, Marti CN, Allen A, Stoddard D, Harmon P (2015). Effectiveness of a school- and community-based academic asthma
health education program on use of effective asthma self-care behaviors in
older school-age students. J Spec Pediatr Nurs.

[34] Kintner EK, Cook G, Marti CN, Gomes M, Meeder L, Van Egeren LA (2015). Effectiveness of a school-based academic asthma health education
and counseling program on fostering acceptance of asthma in older school-age
students with asthma. J Spec Pediatr Nurs.

[35] Nieri T, Apkarian J, Kulis S, Marsiglia FF (2015). Effects of a youth substance use prevention program on stealing,
fighting, and weapon use. J Prim Prev.

[36] Thakore RV, Apfeld JC, Johnson RK, Sathiyakumar V, Jahangir AA, Sethi MK (2015). School-based violence prevention strategy a pilot
evaluation. J Inj Violence Res.

[37] Vander Ploeg KA, Maximova K, McGavock J, Davis W, Veugelers P (2014). Do school-based physical activity interventions increase or
reduce inequalities in health. Soc Sci Med.

[38] Stölzel F, Seidel N, Uhmann S, Baumann M, Berth H, Hoyer J (2014). Be smart against cancer A school-based program covering
cancer-related risk behavior. BMC Public Health.

[39] Wang B, Deveaux L, Knowles V, Koci V, Rolle G, Lunn S (2015). Fidelity of implementation of an evidence-based HIV prevention
program among Bahamian sixth grade students. Prev Sci.

[40] McNaughton DB, Cowell JM, Fogg L (2014). Efficacy of a Latino mother-child communication intervention in
elementary schools. J Sch Nurs.

[41] Pettigrew J, Graham JW, Miller-Day M, Hecht ML, Krieger JL, Shin YJ (2015). Adherence and delivery implementation quality and program
outcomes for the seventh-grade keepin' it REAL program. Prev Sci.

[42] Vander Ploeg KA, McGavock J, Maximova K, Veugelers PJ (2014). School-based health promotion and physical activity during and
after school hours. Pediatrics.

[43] Cao ZJ, Chen Y, Wang SM (2014). Health belief model based evaluation of school health education
programme for injury prevention among high school students in the community
context. BMC Public Health.

[44] Peñalvo JL, Sotos-Prieto M, Santos-Beneit G, Pocock S, Redondo J, Fuster V (2013). The Program SI intervention for enhancing a healthy lifestyle in
preschoolers: first results from a cluster randomized trial. BMC Public Health.

[45] D'Haese S, Van Dyck D, De Bourdeaudhuij I, Cardon G (2013). Effectiveness and feasibility of lowering playground density
during recess to promote physical activity and decrease sedentary time at
primary school. BMC Public Health.

[46] George MW, Trumpeter NN, Wilson DK, McDaniel HL, Schiele B, Prinz R (2014). Feasibility and preliminary outcomes from a pilot study of an
integrated health-mental health promotion program in school mental health
services. Fam Comm Health.

[47] Johnson C, Majzoub K, Lyons S, Martirosyan K, Tattersall P (2016). Eyes That Thrive in School A Program to Support Vision Treatment
Plans at School. J Sch Health.

[48] Hobday K, Ramke J, du Toit R, Pereira SM (2015). Healthy Eyes in Schools An evaluation of a school and
community-based intervention to promote eye health in rural
Timor-Leste. Health Educ J.

[49] Liao LL, Liu CH, Chang FC, Cheng CCJ, Niu YZ, Chang TC (2015). Evaluation of the Health-Promoting School Supporting Network in
Taiwan. J Sch Health.

[50] Totura CM, Figueroa HL, Wharton C, Marsiglia FF (2015). Assessing implementation of evidence-based childhood obesity
prevention strategies in schools. Prev Med Rep.

[51] Flynn A, Zackula R, Klaus NM, McGinness L, Carr S, Macaluso M (2016). Student Evaluation of the Yellow Ribbon Suicide Prevention
Program in Midwest Schools. Prim Care Companion CNS Disord.

[52] Moodley N, Gray G, Bertram M (2016). Projected economic evaluation of the national implementation of a
hypothetical HIV vaccination program among adolescents in South Africa,
2012. BMC Public Health.

[53] Medeiros PF, Cruz JI, R Schneider D, Sanudo A, Sanchez ZM (2016). Process evaluation of the implementation of the Unplugged Program
for drug use prevention in Brazilian schools. Subst Abuse Treat Prev Policy.

[54] Safdie M, Cargo M, Richard L, Lévesque L (2014). An ecological and theoretical deconstruction of a school-based
obesity prevention program in Mexico. Int J Behav Nutr Phys Act.

[55] Garmy P, Jakobsson U, Carlsson KS, Berg A, Clausson EK (2015). Evaluation of a school-based program aimed at preventing
depressive symptoms in adolescents. J Sch Nurs.

[56] Volpe SL, Hall WJ, Steckler A, Schneider M, Thompson D, Mobley C (2013). Process evaluation results from the healthy nutrition
intervention to modify the total school food environment. Health Educ Res.

[57] Chittleborough CR, Nicholson AL, Young E, Bell S, Campbell R (2013). Implementation of an educational intervention to improve hand
washing in primary schools: process evaluation within a randomised
controlled trial. BMC Public Health.

[58] Tahlil T, Coveney J, Woodman RJ, Ward PR (2013). Exploring recommendations for an effective smoking prevention
program for indonesian adolescents. Asian Pac J Cancer Prev.

[59] Clarke AM, Bunting B, Barry MM (2014). Evaluating the implementation of a school-based emotional
well-being programme a cluster randomized controlled trial of Zippy's
Friends for children in disadvantaged primary schools. Health Educ Res.

[60] Bonita S, Wang B, Deveaux L, Lunn S, Rolle G, Mortimer A (2015). Teachers' Patterns of Implementation of an Evidence-Based
Intervention and Their Impact on Student Outcomes Results from a Nationwide
Dissemination over 24-Months Follow-Up. AIDS Behav.

[61] Dinkel DM, Huberty J, Beets MW (2015). Qualitative Evaluation of GoGirlGo Insights From Staff on Using a
Curriculum Within After-School Programs to Improve Physical
Activity. Health Promot Pract.

[62] Cerni OE, Zadro K, Batic-Mujanovic O, Zalihic A (2013). Satisfaction with the program of school bullying prevention and
mental health promotion - cross sectional study among primary school pupils
in Mostar. Acta Med Acad.

[63] Wang LY, Vernon-Smiley M, Gapinski MA, Desisto M, Maughan E, Sheetz A (2014). Cost-benefit study of school nursing services. JAMA Pediatr.

[64] Wang H, Li T, Siahpush M, Chen LW, Huberty J (2017). Cost-Effectiveness of Ready for Recess to Promote Physical
Activity in Children. J Sch Health.

[65] Barron IG, Topping KJ (2013). Exploratory Evaluation of a School-Based Child Sexual Abuse
Prevention Program. J Child Sex Abus.

[66] Safdie M, Jennings-Aburto N, Lévesque L, Janssen I, Campirano-Núñez F, López-Olmedo N (2013). Impact of a school-based intervention program on obesity risk
factors in Mexican children. Salud Publica Mex.

[67] Velsor-Friedrich B, Richards M, Militello LK, Dean KC, Scott D, Gross IM (2015). The Impact of Community Violence on School-Based
Research. J Sch Nurs.

[68] Plaspohl SS, Dixon BT, Streater JA, Hausauer ET, Newman CP, Vogel RL (2013). Impact of School Flu Vaccine Program on Student
Absences. J Sch Nurs.

[69] McIsaac JL, Chu YL, Blanchard C, Rossiter M, Williams P, Raine K (2015). The impact of school policies and practices on students' diets,
physical activity levels and body weights A province-wide practicebased
evaluation. Can J Public Health.

[70] Espelage DL, Low S, Polanin JR, Brown EC (2013). The impact of a middle school program to reduce aggression,
victimization, and sexual violence. J Adolesc Health.

[71] Lubans DR, Smith JJ, Plotnikoff RC, Dally KA, Okely AD, Salmon J (2016). Assessing the sustained impact of a school-based obesity
prevention program for adolescent boys the ATLAS cluster randomized
controlled trial. Int J Behav Nutr Phys Act.

[72] Tomczyk S, Hanewinkel R, Isensee B (2015). 'Klar bleiben': a school-based alcohol prevention programme for
German adolescents-study protocol for a cluster randomised controlled
trial. BMJ Open.

[73] Melnyk BM, Jacobson D, Kelly SA, Belyea MJ, Shaibi GQ, Small L (2015). Twelve-Month Effects of the COPE Healthy Lifestyles TEEN Program
on Overweight and Depressive Symptoms in High School
Adolescents. J Sch Health.

[74] Corriveau N, Eagle T, Jiang Q, Rogers R, Gurm R, Aaronson S (2015). Sustained Benefit Over Four-Year Follow-Up of Michigan's Project
Healthy Schools. Am J Public Health.

[75] Thompson HR, Vittinghoff E, Linchey JK, Madsen KA (2015). Public Disclosure to Improve Physical Education in an Urban
School District Results From a 2-Year Quasi-Experimental
Study. J Sch Health.

[76] Mathews C, Eggers SM, de Vries PJ, Mason-Jones AJ, Townsend L, Aarø LE (2015). Reaching the hard to reach longitudinal investigation of
adolescents' attendance at an after-school sexual and reproductive health
programme in Western Cape, South Africa. BMC Public Health.

[77] Beets MW, Weaver RG, Turner-McGrievy G, Huberty J, Ward DS, Pate RR (2015). Making policy practice in afterschool programs a randomized
controlled trial on physical activity changes. Am J Prev Med.

[78] Madsen KA, Cotterman C, Crawford P, Stevelos J, Archibald A (2015). Effect of the Healthy Schools Program on prevalence of overweight
and obesity in California schools, 2006-2012. Prev Chronic Dis.

[79] Trigwell J, McGee CE, Murphy RC, Porcellato LA, Ussher M, Garnham-Lee K (2015). Process evaluation of a sport-for-health intervention to prevent
smoking amongst primary school children SmokeFree Sports. BMC Public Health.

[80] Wing YK, Chan NY, Man Yu MW, Lam SP, Zhang J, Li SX (2015). A school-based sleep education program for adolescents a cluster
randomized trial. Pediatrics.

[81] Guidry VT, Lowman A, Hall D, Baron D, Wing S (2014). Challenges and benefits of conducting environmental justice
research in a school setting. New Solut.

[82] Charafeddine L, Rafei RE, Azizi S, Sinno D, Alamiddine K, Howson CP (2014). Improving awareness of preconception health among adolescents
experience of a school-based intervention in Lebanon. BMC Public Health.

[83] Shochet I, Montague R, Smith C, Dadds M (2014). A qualitative investigation of adolescents' perceived mechanisms
of change from a universal school-based depression prevention
program. Int J Environ Res Public Health.

[84] Kaufman CE, Whitesell NR, Keane EM, Desserich JA, Giago C, Sam A (2014). Effectiveness of Circle of Life, an HIV-preventive intervention
for American Indian middle school youths a group randomized trial in a
Northern Plains tribe. Am J Public Health.

[85] Jennings JM, Howard S, Perotte CL (2014). Effects of a school-based sexuality education program on peer
educators the Teen PEP model. Health Educ Res.

[86] Takeuchi R, Boureima D, Mizuguchi D, Awazawa T, Kato Y, Akiyama T (2013). Self-assessed approach to improving school health in
Niger. Rural Remote Health.

[87] Demissie Z, Brener N (2017). Demographic Differences in District-Level Policies Related to
School Mental Health and Social Services-United States, 2012. J Sch Health.

[88] Waqa G, Moodie M, Schultz J, Swinburn B (2013). Process evaluation of a community-based intervention program
Healthy Youth Healthy Communities, an adolescent obesity prevention project
in Fiji. Glob Health Promot.

[89] Sebire SJ, Edwards MJ, Kesten JM, May T, Banfield KJ, Bird EL (2016). Process evaluation of the Bristol girls dance
project. BMC Public Health.

[90] Kheirouri S, Alizadeh M (2014). Process evaluation of a national school-based iron
supplementation program for adolescent girls in Iran. BMC Public Health.

[91] Al-Iryani B, Basaleem H, Al-Sakkaf K, Kok G, Borne BVD (2013). Process evaluation of school-based peer education for HIV
prevention among Yemeni adolescentes. SAHARA J.

[92] Stubbs BW, Panozzo CA, Moss JL, Reiter PL, Whitesell DH, Brewer NT (2014). Evaluation of an intervention providing HPV vaccine in
schools. Am J Health Behav.

[93] Montenegro E, Salinas J, Parra M, Lera L, Vio F (2014). Evaluation of a nutrition education intervention in teachers and
students in preschool and primary schools in los Andes,
Chile. Arch Latinoam Nutr.

[94] Bannink R, Broeren S, Heydelberg J, Klooster E, Baar C, Raat H (2014). Your Health, an intervention at senior vocational schools to
promote adolescents' health and health behaviors. Health Educ Res.

[95] Parsons WG, Garcia GM, Hoffman PK (2013). Evaluating School Wellness Policy in Curbing Childhood Obesity in
Anchorage, Alaska. J Sch Nurs.

[96] Melnyk MB, Kelly S, LusK P (2014). Outcomes and Feasibility of a Manualized Cognitive-Behavioral
Skills Building Intervention Group COPE for Depressed and Anxious
Adolescents in School Settings. J Child Adolesc Psychiatr Nurs.

[97] Konishi C, Saewyc E, Homma Y, Poon C (2013). Population-level evaluation of school-based interventions to
prevent problem substance use among gay, lesbian and bisexual adolescents in
Canada. Prev Med.

[98] Lehna C, Todd JA, Keller R, Presley L, Jackson J, Davis S (2013). Nursing students practice primary fire prevention. Burns.

[99] Mmbaga EJ, Kajula L, Aaro LE, Kilonzo M, Wubs AG, Eggers SM (2017). Effect of the PREPARE intervention on sexual initiation and
condom use among adolescents aged 12-4 a cluster randomised controlled trial
in Dar es Salaam, Tanzania. BMC Public Health.

[100] Kiviruusu O, Björklund K, Koskinen HL, Liski A, Lindblom J, Kuoppamäki H (2016). Short-term effects of the "Together at School" intervention
program on children's socio-emotional skills a cluster randomized controlled
trial. BMC Psychol.

[101] Gunawardena N, Kurotani K, Indrawansa S, Nonaka D, Mizoue T, Samarasinghe D (2016). School-based intervention to enable school children to act as
change agents on weight, physical activity and diet of their mothers a
cluster randomized controlled trial. Int J Behav Nutr Phys Act.

[102] Bavarian N, Lewis KM, Acock A, DuBois DL, Yan Z, Vuchinich S (2016). Effects of a School-Based Social-Emotional and Character
Development Program on Health Behaviors A Matched-Pair, Cluster-Randomized
Controlled Trial. J Prim Prev.

[103] Heo M, Irvin E, Ostrovsky N, Isasi C, Blank AE, Lounsbury DW (2016). Behaviors and Knowledge of HealthCorps New York City High School
Students Nutrition, Mental Health, and Physical Activity. J Sch Health.

[104] Dahlqvist HZ, Landstedt E, Gådin KG (2015). What students do schools allocate to a cognitive-behavioural
intervention Characteristics of adolescent participants in Northern
Sweden. Int J Circumpolar Health.

[105] Tahlil T, Woodman RJ, Coveney J, Ward PR (2015). Six-months follow-up of a cluster randomized trial of
school-based smoking prevention education programs in Aceh,
Indonesia. BMC Public Health.

[106] Serafini K, Shipley L, Stewart DG (2015). Motivation and substance use outcomes among adolescents in a
school-based intervention. Addict Behav.

[107] Glang AE, Koester MC, Chesnutt JC, Gioia GA, McAvoy K, Marshall S (2015). The effectiveness of a web-based resource in improving
postconcussion management in high schools. J Adolesc Health.

[108] Marsiglia FF, Kulis SS, Booth JM, Nuño-Gutierrez BL, Robbins DE (2015). Long-term effects of the keepin' it REAL model program in Mexico
substance use trajectories of Guadalajara middle school
students. J Prim Prev.

[109] Kobel S, Wirt T, Schreiber A, Kesztyüs D, Kettner S, Erkelenz N (2014). Intervention effects of a school-based health promotion programme
on obesity related behavioural outcomes. J Obes.

[110] Sharpe H, Schober I, Treasure J, Schmidt U (2013). Feasibility, acceptability and efficacy of a school-based
prevention programme for eating disorders cluster randomised controlled
trial. Br J Psychiatry.

[111] Gesell SB, Sommer EC, Lambert EW, Vides AAR, Whitaker L, Davis L (2013). Comparative effectiveness of after-school programs to increase
physical activity. J Obes.

[112] Andrews JA, Gordon JS, Hampson SH, Gunn B, Christiansen SM, Slovic P (2014). Long-term efficacy of click city(r) tobacco: a school-based
tobacco prevention program. Nicotine Tob Res.

[113] Standage M, Cumming SP, Gillison FB (2013). A cluster randomized controlled trial of the be the best you can
be intervention effects on the psychological and physical well-being of
school children. BMC Public Health.

[114] McNaughton DB, Cowell JM, Fogg L (2014). Adaptation and feasibility of a communication intervention for
Mexican immigrant mothers and children in a school setting. J Sch Nurs.

[115] Tahlil T, Woodman RJ, Coveney J, Ward PR (2013). The impact of education programs on smoking prevention a
randomized controlled trial among 11 to 14 year olds in Aceh,
Indonesia. BMC Public Health.

[116] Monse B, Benzian H, Naliponguit E, Belizario V, Schratz A, Van PHW (2013). The Fit for School Health Outcome Study - a longitudinal survey
to assess health impacts of an integrated school health programme in the
Philippines. BMC Public Health.

[117] Kyle RG, Forbat L, Rauchhaus P, Hubbard G (2013). Increased cancer awareness among British adolescents after a
school-based educational intervention a controlled before-and-after study
with 6-month follow-up. BMC Public Health.

[118] Minary L, Cambon L, Martini H, Wirth N, Acouetey DS, Thouvenot F (2013). Efficacy of a smoking cessation program in a population of
adolescent smokers in vocational schools a public health evaluative
controlled study. BMC Public Health.

[119] Schneider M, DeBar L, Calingo A, Hall W, Hindes K, Sleigh A (2013). The effect of a communications campaign on middle school
students' nutrition and physical activity results of the HEALTHY
study. J Health Commun.

[120] Pbert L, Druker S, Gapinski MA, Gellar L, Magner R, Reed G (2013). A school nurse-delivered intervention for overweight and obese
adolescents. J Sch Health.

[121] Safdie M, Lévesque L, González CI, Salvo D, Islas A, Hernández CS (2013). Promoting healthful diet and physical activity in the Mexican
school system for the prevention of obesity in children. Salud Publica Mex.

[122] Zhang C, Mosa AJ, Hayward AS, Matthews SA (2013). Promoting clean hands among children in Uganda a school-based
intervention using 'tippy-taps'. Public. Health.

[123] Oscós-Sánchez MÁ, Lesser J, Oscós-Flores LD (2013). High school students in a health career promotion program report
fewer acts of aggression and violence. J Adolesc Health.

[124] Isensee B, Hansen J, Maruska K, Hanewinkel R (2016). Effects of a school-based prevention programme on smoking in
early adolescence: a 6-month follow-up of the 'Eigenstandig werden' cluster
randomised trial. BMJ Open.

[125] Cunningham-Sabo L, Lohse B (2013). Cooking with Kids positively affects fourth graders' vegetable
preferences and attitudes and self-efficacy for food and
cooking. Child Obes.

[126] Kansagra SM, Papadouka V, Geevarughese A, Hansen MA, Konty KJ, Zucker JR (2014). Reaching children never previously vaccinated for influenza
through a school-located vaccination program. Am J Public Health.

[127] Ramos P, Pasarín MI, Artazcoz L, Díez E, Juárez O, González I (2013). Healthy and participative schools evaluation of a public health
strategy. Gac Sanit.

[128] Alvirde U, Aguilar SCA, Gómez PFJ, Henao MS, Rodríguez AJG (2013). Results of a community based life style intervention program for
children. Salud Publica Mex.

[129] Serowoky ML, George N, Yarandi H (2015). Using the Program Logic Model to Evaluate ¡Cuídate : A Sexual
Health Program for Latino Adolescents in a School-Based Health
Center. Worldviews Evid Based Nurs.

[130] Nabors L, Burbage M, Woodson KD, Swoboda C (2015). Implementation of an after-school obesity prevention program
helping young children toward improved health. Issues Compr Pediatr Nurs.

[131] Sidhu AK, Sussman S, Tewari A, Bassi S, Arora M (2016). Project EX-India A classroom-based tobacco use prevention and
cessation intervention program. Addict Behav.

[132] Biddle VS, Kern J, Brent DA, Thurkettle MA, Puskar KR, Sekula L (2014). Student assistance program outcomes for students at risk for
suicide. J Sch Nurs.

[133] Primack BA, Douglas EL, Land SR, Miller E, Fine MJ (2014). Comparison of media literacy and usual education to prevent
tobacco use a cluster-randomized trial. J Sch Health.

[134] Sussman AL, Montoya C, Werder O, Davis S, Wallerstein N, Kong AS (2014). Evaluation of an intervention providing HPV vaccine in
schools. Am J Health Behav.

[135] Drubin DG, Kellogg DR (2012). English as the universal language of science opportunities and
challenges. Mol Biol Cell.

[136] Tanaka OY, Tamaki EM (2012). The role of evaluation in decision-making in the management of
health services. Cien Saúde Coletiva.

[137] Brousselle A, Champagne F (2011). Program theory evaluation Logic analysis. Eval Program Plann.

[138] Furtado JP, Vieira-da-Silva LM (2014). The evaluation of health programs and services in Brazil as a
space for knowledge and practice. Cad Saúde Pública.

[139] Viacava F, Ugá MAD, Porto S, Laguardia J, Moreira RS (2012). Evaluation of performance of health systems a model for
analysis. Cienc Saúde Coletiva.

[140] McIsaac J-LD, Penney TL, Ata N, Munro-Sigfridson L, Cunningham J, Veugelers PJ (2017). Evaluation of a health promoting schools program in a school
board in Nova Scotia, Canada. Prev Med Rep.

[141] Langford R, Bonell C, Jones H, Pouliou T, Murphy S, Waters E (2015). The World Health Organization's Health Promoting Schools
framework a Cochrane systematic review and meta-analysis. BMC Public Health.

[142] Kontak JCH, McIsaac J-LD, Penney TL, Kuhle S, Kirk SFL (2017). The picture of health examining school-based health environments
through photographs. Health Promot Int.

